# Primary Diffuse Large B Cell Lymphoma of the Cranial Vault

**DOI:** 10.5812/iranjradiol.7734

**Published:** 2012-06-30

**Authors:** Kiara Rezaei-Kalantari, Kaveh Samimi, Maryam Jafari, Mohammad Ali Karimi, Keyvan Ansari, Mohammad Davoodi, Mahtab Nabi-Meybodi, Mehrnoush Gorjian

**Affiliations:** 1 Department of Radiology, Hazrat Rasoul-e-Akram Hospital, Tehran University of Medical Sciences, Tehran, Iran; 2 Advanced Diagnostic and Interventional Radiology Research Center (ADIR), Tehran University of Medical Sciences, Tehran, Iran; 3 Department of Radiology, Loghman Hakim Hospital, Shahid Beheshti University of Medical Sciences, Tehran, Iran; 4 Hazrat Rasoul-e-Akram Hospital, Tehran University of Medical Sciences, Tehran, Iran

**Keywords:** Lymphoma, B-Cell, Dura Mater, Cranial Vault

## Abstract

Primary non-Hodgkin’s lymphoma of the cranial vault is extremely rare. This case report presents a 42-year-old man with a painless subcutaneous scalp mass which extended intracranially associated with recent mild headache. Initial computed tomography and magnetic resonance imaging revealed two lesions emanating from the skull. Biopsy revealed a diagnosis of diffuse large B cell lymphoma (DLBCL). A thorough work-up revealed no other point of involvement. This case is concerned about considering lymphoma in the differential diagnosis of calvarial lesions with both intra- and extra cranial extensions but without obvious intervening bony destruction.

## 1. Introduction

Although malignant lymphoma of the central nervous system has become common due to progressively increasing immunosuppressive conditions, a primary malignant lymphoma particularly diffuse large B cell lymphoma (DLBCL) of the dura and/or cranial vault is a rare occurrence and has been described as case reports. The overall rate for diffuse large cell lymphomas (DLCLs) is approximately 4.68 cases per 100,000 people per year (1, 2, 3, 4). These tumors may extend into both the brain and the scalp, leading to both extracranial and intracranial components mostly mimicking meningioma (5). The authors report a patient with isolated DLBCL of the cranial vault, presenting as a left frontal skull lesion beginning from 4 months before.

## 2. Case Report

A 42-year-old man with a left frontal head lump which had been largening progressively during the past 4 months came to our hospital. He also complained of a recent headache. There was no significant medical history including trauma or seizure. Local examination revealed a non-tender soft 5 × 10 cm sized swelling over the left frontal region, fixed to the underlying skull. There was no sign of inflammation or tenderness over the lesion. In general examination, no focal neurological deficit, lymphadenopathy or organomegaly was noted.

**Figure 1:**
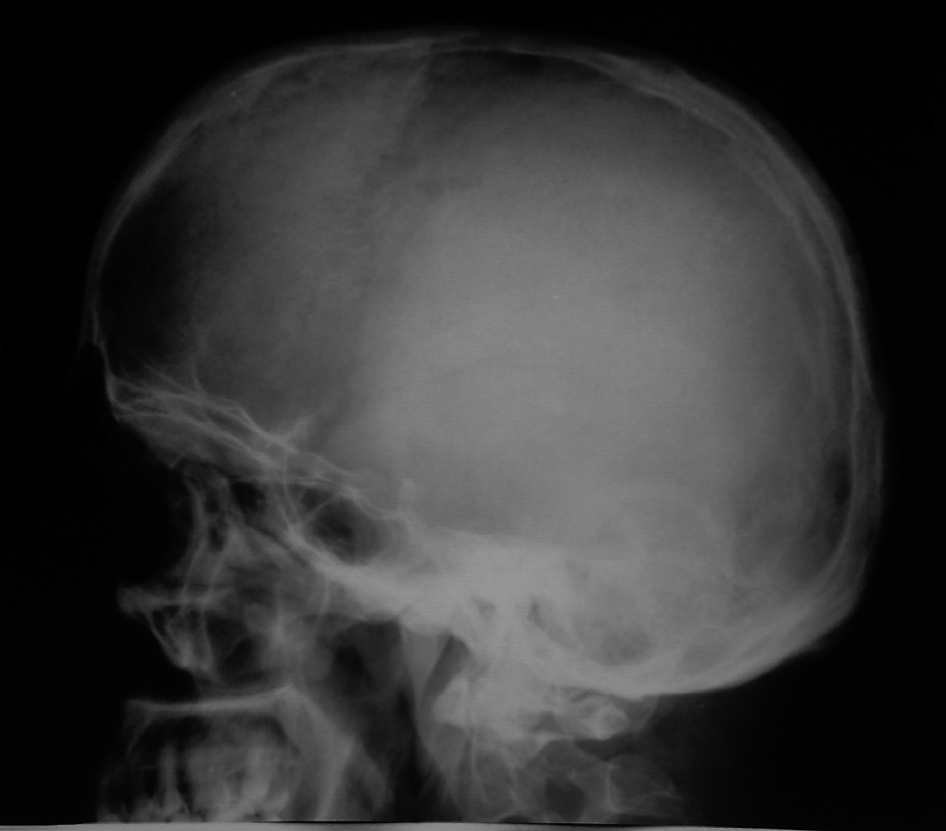
A round fairly well-defined soft tissue density superimposed on the frontal skull without considerable underlying bony destruction.

**Figure 2:**
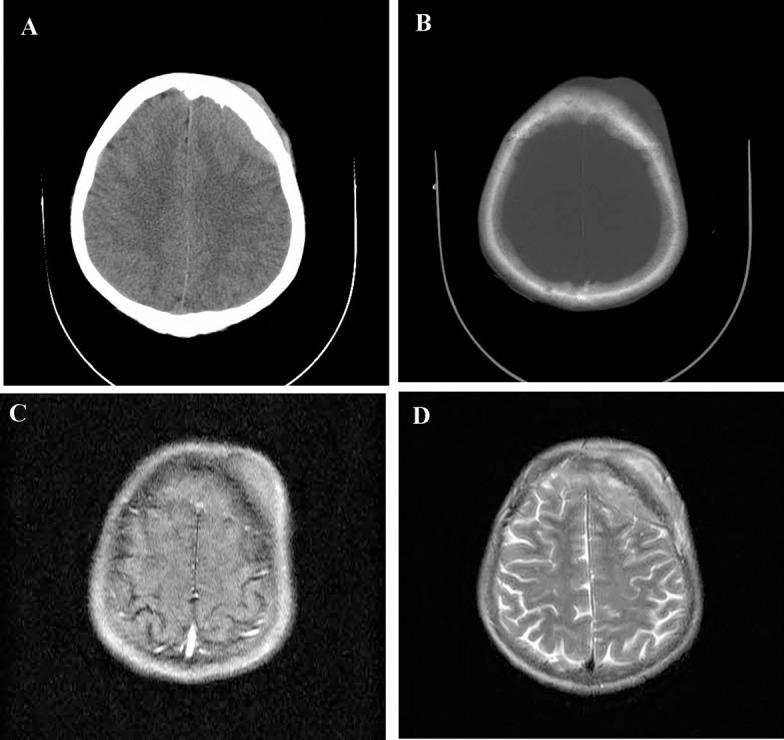
Axial CT scan (A,B), a calvarial lesion with both extra- and intracranial isodense soft tissue components with mild calvarial hyperostotic changes. T1-weighted (C) and T2 weighted (D) MR images show the lesion nearly isointense to gray matter.

**Figure 3:**
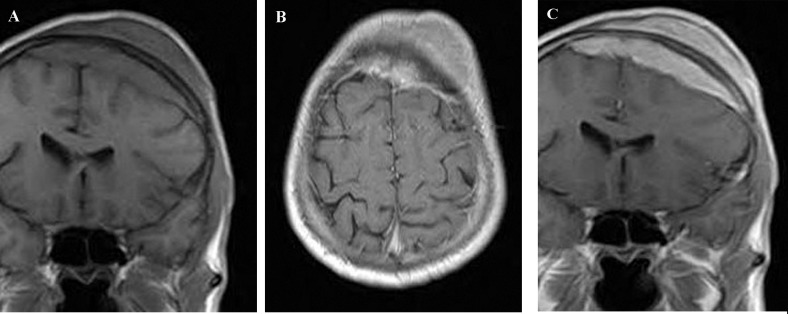
T1-weighted MR image before (A) and after (B,C) contrast medium reveals marked enhancement of the lesion and the dura beneath. Note also the almost normal marrow signal of the intervening skull in the regions without hyperostosis

**Figure 4:**
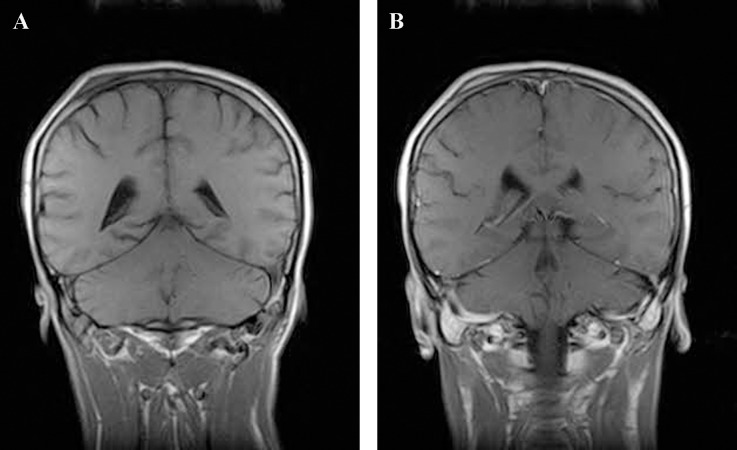
T1-weighted coronal MR image before (A) and after (B) gadolinium. A second small lesion with the same MR signal characteristics extending outward from the right parietal bone

Skull radiographs showed a relatively well-defined soft tissue density over the left frontal bone, without evidence of calcification, or obvious bone erosion or sclerosis (Figure 1). Noncontrast CT demonstrated a slightly hyper-dense mass emanating from the calvaria extending both intracranially and extracranially with minimal underlying morphological changes in the bone as mild increase in bone thickness (hyperostosis) (Figure 2). On MRI, T1 and T2-weighted images revealed lesion isointensity to gray matter (Figure 3A, B). Following gadolinium injection, intense homogeneous enhancement of the lesion and the dural tail beneath was noted (Figure 4). In addition, another small mass was detected on the more caudal right parietal bone. This mass was completely extracranial and had the same MR signal characteristics. The preoperative impression of meningioma with transcalvarial extension was made. Slight changes in the bone signal in comparison to uninvolved regions were also seen (Figures 2 and 3).

**Figure 5:**
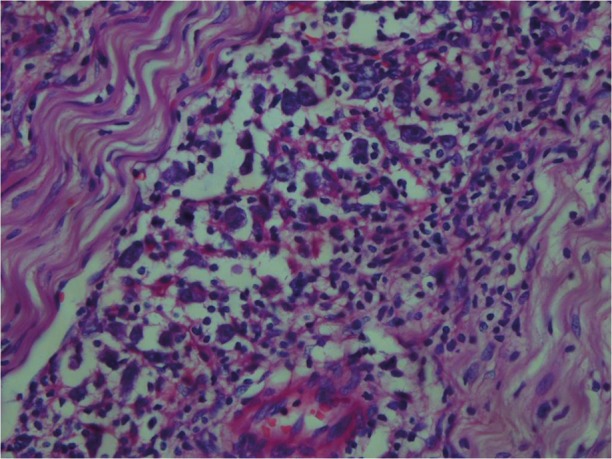
Infiltration of meningial tissue by lymphoid cells. Note proliferation of large atypical cells with prominent eosinophilic nucleoli mixed with lymphocytes.

**Figure 6:**
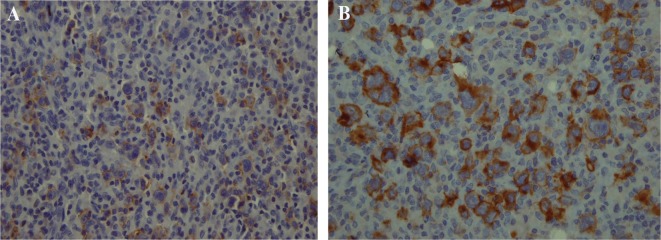
Positive staining for CD20 (A) and CD30 (B) in the large cells could be seen.

The histological examination of the surgical specimen showed lymphoid infiltration of the skull bone and adjacent dura, with tumor cells demonstrating large vesicular nuclei and extremely large reddish nucleoli as well as scattered mitotic figures (Figure 5). Immunohistochemistry revealed positive staining for CD20 and CD30 and negative staining for ALK (Figure 6). The resultant diagnosis of diffuse large B-cell lymphoma with anaplastic features was reported. A thorough search in order to find another source of involvement was made. Complete blood cell count, biochemical profiles and viral markers including HIV were unremarkable. The CSF cytology examination was negative for malignant cells. The bone marrow biopsy results were free of tumor. Contrast-enhanced CT (CECT) scan of the chest, abdomen and pelvis were negative for any considerable finding. So, the diagnosis of primary DLBCL of the cranial vault was established.

## 3. Discussion 

Cranial vault lymphoma is more often associated with intra- and extracranial extension and intervening skull bone destruction (6-8). In our case, the evidences of skull destruction were not well apparent on imaging, but confirmed by histopathology. Imaging characteristics of cranial lymphoma are nonspecific. Most reported cases, as our case, were isointense on nonenhanced MR images to gray matter with avid post-contrast enhancement (9). Our patient had two soft tissue lesions; one with both intra- and extracranial components and the other with just an extracranial extension. We found no other report of two adjacent lymphomatous soft tissue masses emanating from the skull especially only one with an extracranial outspread.

Regarding gender and the involved region, our case is similar to other cases reported in the literature (9, 10, 11, 12). But comparing the age, this case presented at least two decades earlier than the other before. The differential diagnosis of tumors arising from the calvarium and extending both intra- and extracranially consist of metastatic or primary skull tumors and intraosseous meningioma (6). However, these lesions commonly invade and destruct the interposed skull bone. Only a few cases of scalp and intracranial involvement without a sign of intervening bone destruction have been reported. In our case, although microscopic examination revealed lymphoid infiltration, there was only minimal imaging signs of bone involvement. It has been suggested that presence of the tumor on the scalp and dura sides, without skull bone invasion between might be one of the characteristics of lymphoma (5). We did not perform a simple needle biopsy for scalp tumor, instead craniectomy with both scalp bone and intracranial tumor excisions was carried out. As proposed by Ochiai et al. (5), it seems that needle biopsy of the scalp tumor, which does not cause significant neurologic symptoms, may obviate surgery, leading the treatment plan to appropriate radiotherapy and chemotherapy. Treatment of diffuse large cell lymphomas (DL-CLs) is primarily with cytotoxic agents, with or without radiation therapy. The role of surgery in these tumors is limited. The cyclophosphamide, adriamycin, vincristine, prednisone (CHOP) regimen was among the first combinations to produce complete response and long-term survival. For patients with advanced DLCL, another standard therapy exists: the addition of a monoclonal antibody against CD20 (rituximab) to CHOP. In addition, high-dose chemotherapy in the setting of stem cell/bone marrow transplantation has become a useful treatment modality in the management of this disease. The presented patient is under CHOP regimen and has received 2 cycles of CHOP and his headache has reduced. Treatment plan include four cycles of CHOP and then involved-field radiation therapy.
